# Gender, race and socioeconomic influence on diagnosis and treatment of thyroid disorders in the Brazilian Longitudinal Study of Adult Health (ELSA-Brasil)

**DOI:** 10.1590/1414-431X20154445

**Published:** 2015-06-23

**Authors:** R.D. Olmos, R.C. de Figueiredo, E.M. Aquino, P.A. Lotufo, I.M. Bensenor

**Affiliations:** 1 Hospital Universitário, Universidade de São Paulo, São Paulo, SP, Brasil; 2 Faculdade de Medicina, Universidade de São Paulo, São Paulo, SP, Brasil; 3 Universidade Federal de São João Del-Rei,, São João Del-Rei, MG, Brasil; 4 Instituto de Saúde Coletiva, Universidade Federal da Bahia, Salvador, BA, Brasil

**Keywords:** Thyroid dysfunction, Treatment of thyroid disorders, Levothyroxine, Gender, Socioeconomic status, Race

## Abstract

Thyroid diseases are common, and use of levothyroxine is increasing worldwide. We investigated the influence of gender, race and socioeconomic status on the diagnosis and treatment of thyroid disorders using data from the Brazilian Longitudinal Study of Adult Health (ELSA-Brasil), a multicenter cohort study of civil servants (35-74 years of age) from six Brazilian cities. Diagnosis of thyroid dysfunction was by thyrotropin (TSH), and free thyroxine (FT4) if TSH was altered, and the use of specific medications. Multivariate logistic regression models were constructed using overt hyperthyroidism/hypothyroidism and levothyroxine use as dependent variables and sociodemographic characteristics as independent variables. The frequencies of overt hyper- and hypothyroidism were 0.7 and 7.4%, respectively. Using whites as the reference ethnicity, brown, and black race were protective for overt hypothyroidism (OR=0.76, 95%CI=0.64-0.89, and OR=0.53, 95%CI=0.43-0.67, respectively, and black race was associated with overt hyperthyroidism (OR=1.82, 95%CI=1.06-3.11). Frequency of hypothyroidism treatment was higher in women, browns, highly educated participants and those with high net family incomes. After multivariate adjustment, levothyroxine use was associated with female gender (OR=6.06, 95%CI=3.19-11.49) and high net family income (OR=3.23, 95%CI=1.02-10.23). Frequency of hyperthyroidism treatment was higher in older than in younger individuals. Sociodemographic factors strongly influenced the diagnosis and treatment of thyroid disorders, including the use of levothyroxine.

## Introduction

Thyroid diseases are very common in middle-aged and older adults (1). Hypothyroidism is one of the diseases most commonly associated with long-term prescription drug use and is the most common hormone deficiency disease; levothyroxine is the treatment of choice (2). Recent data (July 2013 to June 2014) from MEDSCAPE on the use of medications in the USA indicate that levothyroxine was one of the most prescribed drugs, with 22.5 million prescriptions (3). Additional data show that the rate of thyroid hormone prescription has increased in recent years (4,5). Hyperthyroidism occurs less frequently than hypothyroidism, but is symptomatic and requires prompt diagnosis and treatment (6).

Two previous studies in Brazil evaluated the influence of sociodemographic factors on diagnosis and treatment of thyroid disorders. Bensenor et al. (7), in a population-based sample of older men and women (≥65 years of age), reported that 40% of women with thyroid disorders had been diagnosed and were under treatment compared with only 9% of men. In addition, most participants were not aware of the diagnosis of thyroid dysfunction (7). In the second study, Sichieri et al. (8) reported a clear influence of race on prevalence of hypothyroidism, 16.7% in whites, 8.8% in brown, and 6.9% in black women. Compared with brown women, the adjusted odds ratio (OR) for black women was 0.45 [95% confidence interval (CI)=0.30-0.68] and 0.34 (95%CI=0.18-0.63) for brown women (8).

The available data show that the prescribing rates of thyroid hormone in the United States (IMS Institute) and in the United Kingdom have been increasing recently (5,9-11). In the United Kingdom, Taylor et al. (12) reported that the median thyrotropin (TSH) level at the initiation of levothyroxine therapy fell from 8.7 to 7.9 mIU/L between 2001 and 2009. The OR (adjusted for changes in population demographics) for prescribing levothyroxine at a TSH level of 10.0 mIU/L or less in 2009 compared with 2001 was 1.30 (95%CI=1.19-1.42). They also found that older individuals, or individuals diagnosed with cardiac risk factors, were more likely to begin levothyroxine therapy at a thyrotropin level of 10.0 mIU/L or less (12).

In this cross-sectional study, we investigated the influence of sociodemographic characteristics on the diagnosis and treatment of thyroid dysfunction, use of levothyroxine, and associated factors. We analyzed relevant data from the Brazilian Longitudinal Study of Adult Health (ELSA-Brasil), which is a multicenter cohort study of civil servants 35-74 years of age from six Brazilian cities.

## Material and Methods

### Study recruitment

The ELSA-Brasil study has been previously described (13-15). Briefly, 15,105 civil servants 35-74 years of age from six cities in Brazil (Belo Horizonte, Porto Alegre, Rio de Janeiro, Salvador, São Paulo and Vitória) were enrolled between August 2008 and December 2010. The aim of the study was to determine the incidences of cardiovascular diseases, diabetes mellitus, renal failure, cognitive impairment, and the most important associated risk factors. The sample size estimation was calculated to permit gender-specific analyses. The ELSA-Brasil protocol was approved at each of the six study centers by the local Institutional Review Board addressing research in human participants. All participants provided signed informed consent.

### Data collection

Each participant was interviewed at the workplace and again during a visit to the Research Center when they were given a clinical examination following standard protocols developed for the study. Trained study staff conducted the interviews and examinations following strict quality control procedures as previously described (16). The study questionnaire addressed sociodemographic factors including age and gender, the level of formal education, mean monthly income, race/skin color, and marital and smoking status. Educational level was recorded as <9 years, 9-11 years, and >11 years of schooling. Net family income was recorded as ≤US$1245, US$1246-3319, and ≥US$3320. Race/skin color was self-reported as white, brown, black, Asian, and native. Asian and native were combined and reported as “other”. Smoking status was defined as never smoked, former smoker, and current smoker. The usual type of alcohol consumed, frequency of intake, and drinking patterns were recorded and were also categorized as never, past and current (17). All participants were asked about their use of prescription and nonprescription drugs in the previous 2 weeks (17).

Weight was measured with the participant wearing light clothes. Body-mass index was calculated as weight divided by height in meters squared (kg/m^2^). Waist circumference (cm) was measured with a flexible tape midway between the costal arch and femoral iliac crest according to a standardized World Health Organization (WHO) protocol (18). Blood pressure was measured after a 5 min rest; three measurements were taken with an interval of 1 min. The mean of the last two determinations was recorded as systolic and diastolic blood pressure. Participants who reported use of medication to treat arterial hypertension, or presented with a systolic blood pressure ≥140 mmHg or a diastolic blood pressure ≥90 mmHg were considered to have arterial hypertension. Participants who reported a history or use of medication to treat diabetes mellitus, presented with a fasting plasma glucose ≥126 mg/dL (measured only once), or a 2-h plasma glucose ≥200 mg/dL after 75 g of anhydrous glucose, or HbA1C ≥6.5% were considered to have diabetes mellitus. Dyslipidemia was defined by low-density lipoprotein cholesterol (LDL-C) >130 mg/dL or use of a cholesterol lowering medication.

### Definition of thyroid function

#### Thyroid function

Venous blood samples were drawn after an overnight fast, and centrifuged at 2500 *g* for 15 min to obtain serum for biochemistry and determination of hormone levels. TSH and FT4 levels were determined by a third-generation immunoenzymatic assay (Siemens, USA). Thyroid dysfunction was assessed by TSH and FT4 levels or by routine use of thyroid hormones or anti-thyroid medications, such as propylthiouracil or methimazole. Free thyroxine levels were evaluated only in participants found to have altered TSH levels. Therefore, if TSH levels were normal, FT4 levels were not obtained. Cut-off values for TSH and FT4 were similar to those used in the National Health and Nutritional Examination Survey (NHANES) III (19) and recommended by Surks et al. (20). For TSH, they were <0.4 mIU/L for hyperthyroidism and >4.0 mIU/L for hypothyroidism, and for FT4 they were <0.8 ng/dL for hypothyroidism and >1.9 ng/dL for hyperthyroidism. Participants were placed in one of five groups based on TSH and FT4 levels (if TSH was altered) and their use of medications to treat thyroid disorders. These were clinical hyperthyroidism (low TSH, high FT4, or use of medications to treat hyperthyroidism), subclinical hyperthyroidism (low serum TSH, normal FT4, and no use of thyroid drugs), euthyroidism (normal TSH and no use of thyroid drugs), subclinical hypothyroidism (high TSH, normal FT4 levels, and no use of thyroid drugs), and clinical hypothyroidism (high TSH, low FT4, or use of levothyroxine). Therefore, subclinical thyroid disease was a category only for participants not using any drugs for treatment of thyroid disorders. We excluded participants using drugs that can interfere with thyroid function (e.g., amiodarone, carbamazepine, carbidopa, furosemide, haloperidol, heparin, levodopa, lithium, metoclopramide, phenytoin, propranolol, primidone, rifampicin, or valproic acid) (21,22).

Fasting plasma glucose was measured using a hexokinase method (reference values=70-99 mg/dL). Total- and HDL-cholesterol and triglycerides were measured using an enzymatic colorimetric assay (ADVIA Chemistry, Siemens). Reference values were ≤200 mg/dL for total cholesterol, >40 mg/dL for HDL-cholesterol in men and >50 mg/dL in women, and <150 mg/dL for triglycerides. LDL-cholesterol concentration was estimated using the Friedewald equation.

### Statistical analysis

Prevalence of thyroid disease rates and frequency of treatment are reported as number of cases per 100 persons with respective 95% confidence intervals (CIs). Categorical variables are reported as proportions and compared using the chi-square test. Continuous variables are reported as means±standard deviation (SD) and compared using analysis of variance (ANOVA). A logistic regression model was constructed with overt hyperthyroidism and hypothyroidism as dependent variables and sociodemographic characteristics, smoking, alcohol use, and a health insurance plan as independent variables. A second logistic regression model was constructed with levothyroxine as the dependent variable and factors potentially associated with levothyroxine, including gender, race/skin color, education, per capita income (≤US$1245, US$1246-3319, and ≥US$3320), health insurance, and smoking (never, former or current) and alcohol use (never, past or current) as the independent variables. ORs with 95%CI were calculated with multivariate adjustment for all independent variables (age, gender, race/skin color (white, brown, black, Asian, or native), education (<9, 9-11, >11 years of formal education), and net family income (≤US$1245, US$1246-3319, and ≥US$3320). The analyses were done with SPSS version 22.0 (IBM Corp., USA). P values <0.05 were considered to be significant.

## Results

Of the 15,105 participants, 17 were excluded because of missing TSH and FT4 data and 19 for lack of data on medication use. Nine participants were excluded because they presented with low but near normal TSH values and FT4 levels, suggesting central hypothyroidism, and four because they were using levothyroxine and methimazole concurrently. An additional 466 of the remaining 15,056 participants were excluded because they were using medications that can alter thyroid function or interfere with TSH and FT4 assays. Table 1 shows the general characteristics of the remaining 14,590 participants according to the presence or absence of thyroid diseases. As expected, significantly more women than men were diagnosed with overt hypothyroidism (P<0.0001). Participants with overt hypothyroidism had more years of education and higher net family income than the other groups (both P<0.0001). Frequencies of subclinical and overt hypothyroidism were highest in white, intermediate in brown and lowest in black participants (P<0.0001). The frequency of subclinical and overt hyperthyroidism was also higher in whites (although not as high as for hypothyroidism), but frequencies in browns and blacks were similar. The proportions of participants reporting current smoking were higher among those with overt and subclinical hyperthyroidism than in the other study groups (P<0.0001).

**Table:**
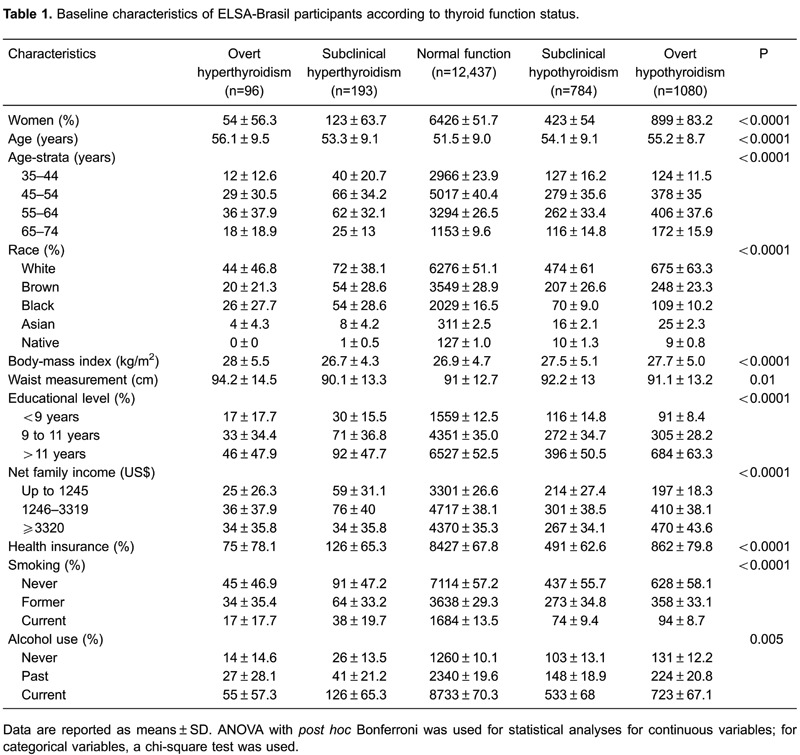

The overall frequency of subclinical hyperthyroidism was 1.3% (95%CI=0-2.9), 0.5% (95%CI=0-2.2) in men, and 0.8% (95%CI=0-2.1) in women. Overt hyperthyroidism was present in 0.7% (95%CI=0-2.4) overall, in 0.3% (95%CI=0-2.0) of men, and 0.4% (95%CI=0-2.1) of women. Subclinical hypothyroidism was present in 5.4% (95%CI=3.8-7.0) overall, 2.5% (95%CI=0.9-4.1) of men, and 2.9% (95%CI=1.3-4.5) of women. Overt hypothyroidism was present in 7.4% (95%CI=5.8-9.0) overall, 1.2% (95%CI=0-2.8) of men, and 6.2% (95%CI=4.6-7.8) of women.

Table 2 shows the ORs of overt hyper- and hypothyroidism according to sociodemographic variables after multivariate adjustment. The frequencies of thyroid diseases were higher in women than in men and increased with age for both hyper- and hypothyroidism. Brown, black and other, i.e., Asian plus native, skin colors were protective for overt hypothyroidism using white as the reference. Black skin color was a risk factor for overt hyperthyroidism also using white as the reference. Overt hyperthyroidism was associated with current smoking, and both subclinical thyroid diseases were associated with having a health insurance plan.

**Table:**
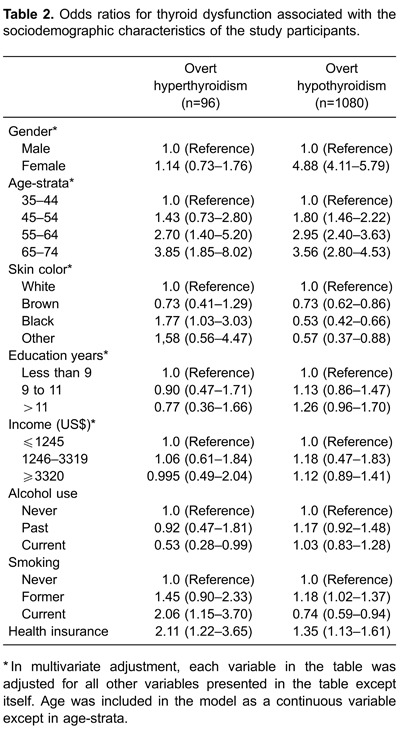

Table 3 shows the frequency of hypothyroidism and hyperthyroidism treatment according to sociodemographic characteristics. The frequency of hypothyroidism treatment was higher in women than in men (97.3% *vs* 87.3%, P<0.0001) and higher in whites than in browns (96.9% *vs* 93.5%) or blacks (96.9% *vs* 91.7%, P=0.03). The frequency of hypothyroidism treatment was higher in highly educated participants (97.7%) than in those with an intermediate level (93.8%), and in those with the least education (86.8%, P<0.0001). The same pattern was seen in those with high (97.7%), intermediate (97.1%), and low (88.3%) net family income (P<0.0001). The only sociodemographic characteristic that was found to influence frequency of hyperthyroidism treatment was age. All participants (100%) 65-74 years of age were being treated compared with only 50% of those who were 35-44 years of age (P<0.0001).

**Table:**
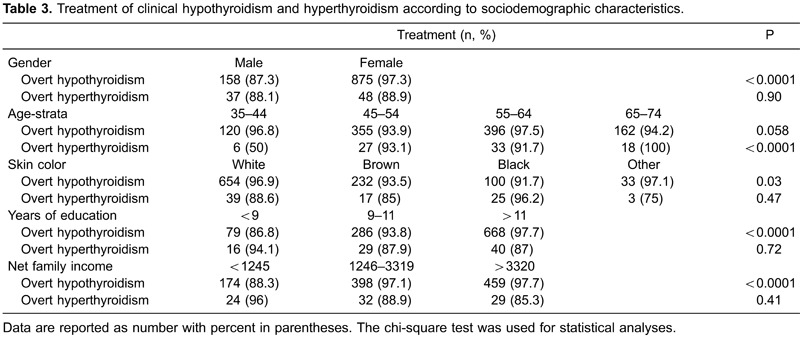

Table 4 shows the association of levothyroxine use with some sociodemographic risk factors after multivariate adjustment for age, gender, race/skin color, education, income per capita, and having a health insurance plan. The reference participants were those not using levothyroxine. The highest ORs for levothyroxine use were female gender (OR=6.28, 95%CI=3.19-12.36) and high net family income (OR=3.07, 95%CI=1.006-9.38).

**Table:**
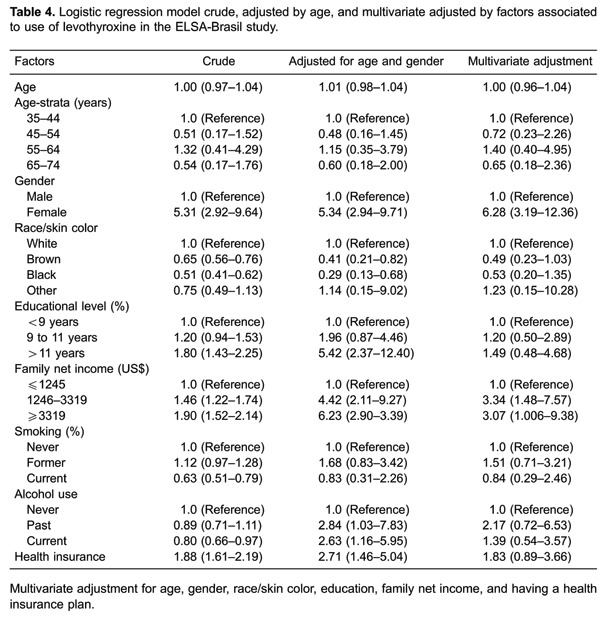

## Discussion

Our results showed high frequencies of thyroid diseases in women and men. Brown and black skin colors were protective for hypothyroidism compared with white, while black skin color was associated with hyperthyroidism. We found that gender, race, and socioeconomic status influenced the diagnosis and treatment of hypothyroidism, with men, browns, blacks, and subjects with low socioeconomic status having lower frequencies of treatment for hypothyroidism. For hyperthyroidism, the only influence on diagnosis and treatment was age, with younger participants (i.e., 35-44 years of age) having a lower frequency of treatment than older participants.

The frequencies of thyroid diseases in this sample were similar to those reported by previous studies in Brazil (7,8). Our data also confirmed previous data that the prevalence of hypothyroidism in Brazil is lower in browns and blacks than in whites, with an intermediate prevalence among brown-skinned people (8). Although that study (8) included only women, in our study, the overall findings for the entire sample were similar to those for men and women evaluated separately. Despite the comparable results, there is a very important difference in the way that race was determined in these two studies. In Sichieri et al. (8), an interviewer placed each of the participants into a race category. In ELSA-Brasil, race was self-reported by participants who chose among five prespecified categories, i.e., white, brown, black, Asian, and Native, as in the Brazilian census. Regardless of how race was classified in these two studies, the results were very similar for protection against hypothyroidism, especially in individuals with black skin color. Contrasting with this data, black race was associated with overt hyperthyroidism. However, the effect of skin color is more pronounced in hypothyroidism than in hyperthyroidism. To the best of our knowledge, this is the first study to demonstrate this association between skin color and hyperthyroidism in the Brazilian population.

We tried to better understand the influences of gender, skin color, and socioeconomic level on the diagnosis and treatment of thyroid diseases. Because thyroid disorders are more frequent in women than men it is likely that physicians screen women more frequently than men, and although guidelines for screening in women have been available since the beginning of this century, there is no consensus (23). Our data revealed a selective effect of screening in women for overt hypothyroidism but not for hyperthyroidism. It is possible that the symptoms of hyperthyroidism are easily detected by physicians regardless of gender, but the symptoms of hypothyroidism might be confounded by age-related symptoms and thus not be obvious. Therefore, the effect of screening in women is clearer for hypothyroidism than for hyperthyroidism. Another important point is that women generally seek medical services more frequently than men for thyroid conditions (24,25) and for other problems (26,27), a pattern that is also true for Brazil (28). Our data showed a higher frequency of treatment for hypothyroidism in whites than in browns and blacks and in participants in high education and income categories compared with less educated and lower income participants. However, after multivariate adjustment the only association with levothyroxine use that remained significant was high income, suggesting that access to health care was the most important influence. Our results differ from those of Bensenor et al. (7) who reported lower frequencies of treatment of hypothyroidism (30%) and of hyperthyroidism (44.4%) in a population-based sample of men and women living in poor neighborhoods. The corresponding treatment rates in this study were 95.6 and 88.5%. Compared with older people living in poor neighborhoods, ELSA-Brasil study participants have a higher income and probably a better access to health care. Skin color and education are likely confounders associated with high income.

Our results also suggest that access to health services was associated with increased use of levothyroxine. In multivariate analysis after adjustment for all confounders, only female gender and high net family income persisted as independent risk factors of levothyroxine. The higher frequency of treatment in subjects with a high socioeconomic position suggests that access to health services was easier in that subgroup. One important study limitation is that we did not know the reason for levothyroxine use and if it was used off-label. Several studies and health statistics have reported an increase in the use of levothyroxine in the United States (4), and the United Kingdom (5,9-11). Reports of an increase in levothyroxine use in Brazil have appeared in recent years in the lay press (29,30).

In contrast to hypothyroidism, the only sociodemographic factor associated with frequency of hyperthyroidism treatment was age. Treatment of hyperthyroidism was less frequent among participants who were 35-44 years of age compared with those who were 45 years of age or older. It is possible that hyperthyroidism symptoms are easier to detect in older people because they contrast with symptoms of ageing.

Our study has some limitations. First, we asked about chronic use of medications only in the previous 2 weeks. Second, the reason for use of levothyroxine was not determined. Third, information about skin color was self-reported. The strength of our data is supported by the nature of ELSA-Brasil, which is a large, multicenter, cohort study with 15,105 participants, and it collects very good information about thyroid dysfunction and treatment in Brazil, a country where such information has been scarce (13).

Our results demonstrate important influences of gender, race, and socioeconomic position on the diagnosis and treatment of thyroid disorders in Brazil. This knowledge should be incorporated in the design of public health policies to be implemented in the near future.

## References

[1] Helfand M, Redfern CC (1998). Clinical guideline, part 2. Screening for thyroid
disease: an update. American College of Physicians. Ann Intern Med.

[2] Roberts CG, Ladenson PW (2004). Hypothyroidism. Lancet.

[3] Use of levothyroxine (2014). http://www.medscape.com/viewarticle/825053.

[4] IMS Institute for Healthcare Informatics (2014). The use of medicines in the United States: review of
2010. http://www.imshealth.com/deployedfiles/imshealth/Global/Content/IMS%20Institute/Static%20File/IHII_UseOfMed_report.pdf.

[5] Mitchell AL, Hickey B, Hickey JL, Pearce SH (2009). Trends in thyroid hormone prescribing and consumption in
the UK. BMC Public Health.

[6] Cooper DS (2003). Hyperthyroidism. Lancet.

[7] Bensenor IM, Goulart AC, Lotufo PA, Menezes PR, Scazufca M (2011). Prevalence of thyroid disorders among older people:
results from the São Paulo Ageing & Health Study. Cad Saúde Pública.

[8] Sichieri R, Baima J, Marante T, de Vasconcellos MT, Moura AS, Vaisman M (2007). Low prevalence of hypothyroidism among black and Mulatto
people in a population-based study of Brazilian women. Clin Endocrinol.

[9] Primary Care: Health & Social Care Information Centre (2014). Prescriptions dispensed in the community: statistics for
England: 2002 to 2012. http://www.ic.nhs.uk/statistics-and-data-collections/primary-care/prescriptions/prescriptions-dispensed-in-the-community-england-statistics-for-2000-to-2010.

[10] Health and Social Care Information Center entitled Prescription Cost
Analysis, England - 2013 [NS] (2014). http://www.hscic.gov.uk/catalogue/PUB13887.

[11] Davies JE, Taylor DG (2013). Individualisation or standardisation: trends in National
Health Service prescription durations in England 1998-2009. Prim Health Care Res Dev.

[12] Taylor PN, Iqbal A, Minassian C, Sayers A, Draman MS, Greenwood R (2014). Falling threshold for treatment of borderline elevated
thyrotropin levels-balancing benefits and risks: evidence from a large
community-based study. JAMA Intern Med.

[13] Aquino EM, Barreto SM, Bensenor IM, Carvalho MS, Chor D, Duncan BB (2012). Brazilian Longitudinal Study of Adult Health
(ELSA-Brasil): objectives and design. Am J Epidemiol.

[14] Lotufo PA (2013). [Setting up the longitudinal study for adult health
(ELSA-Brasil]. Rev Saúde Pública.

[15] Schmidt MI, Duncan BB, Mill JG, Lotufo PA, Chor D, Barreto SM (2015). Cohort Profile: Longitudinal Study of Adult Health
(ELSA-Brasil). Int J Epidemiol.

[16] Bensenor IM, Griep RH, Pinto KA, de Faria CP, Felisbino-Mendes M, Caetano EI (2013). Routines of organization of clinical tests and
interviews in the ELSA-Brasil investigation center. Rev Saude Publica.

[17] Chor D, Alves MG, Giatti L, Cade NV, Nunes MA, Molina MC (2013). [Questionnaire development in ELSA-Brasil: challenges of
a multidimensional instrument]. Rev Saúde Pública.

[18] Lohman TG, Roche AF, Martorell R (1988). Anthropometric standardization reference
manual..

[19] Hollowell JG, Staehling NW, Flanders WD, Hannon WH, Gunter EW, Spencer CA (2002). Serum TSH, T(4), and thyroid antibodies in the United
States population (1988 to 1994): National Health and Nutrition Examination Survey
(NHANES III). J Clin Endocrinol Metab.

[20] Surks MI, Ortiz E, Daniels GH, Sawin CT, Col NF, Cobin RH (2004). Subclinical thyroid disease: scientific review and
guidelines for diagnosis and management. JAMA.

[21] Lai EC, Yang YH, Lin SJ, Hsieh CY (2013). Use of antiepileptic drugs and risk of
hypothyroidism. Pharmacoepidemiol Drug Saf.

[22] Dong BJ (2000). How medications affect thyroid function. West J Med.

[23] Bensenor I (2002). Screening for thyroid disorders in asymptomatic adults
from Brazilian populations. São Paulo Med J.

[24] Canaris GJ, Manowitz NR, Mayor G, Ridgway EC (2000). The Colorado thyroid disease prevalence
study. Arch Intern Med.

[25] Gussekloo J, van Exel E, de Craen AJ, Meinders AE, Frolich M, Westendorp RG (2004). Thyroid status, disability and cognitive function, and
survival in old age. JAMA.

[26] Stoverinck MJ, Lagro-Janssen AL, Weel CV (1996). Sex differences in health problems, diagnostic testing,
and referral in primary care. J Fam Pract.

[27] Koutis AD, Isacsson A, Lindholm LH, Lionis CD, Svenninger K, Fioretos M (1991). Use of primary health care in Spili, Crete, and in
Dalby, Sweden. Scand J Prim Health Care.

[28] Aquino EM, Menezes GM, Amoedo MB (1992). [Gender and health in Brazil: considerations based on
the National Household Sampling Survey]. Rev Saúde Pública.

[29] Uso de hormônios (2014). http://oglobo.globo.com/sociedade/saude/hormonio-da-tireoide-tem-onda-de-consumo-para-emagrecimento-7339685.

[30] Hormônios da tireoide (2014). http://veja.abril.com.br/noticia/saude/venda-de-hormonios-sinteticos-da-tireoide-cresce-65-em-quatro-anos.

